# Fast synthesis of high surface area bio-based porous carbons for organic pollutant removal

**DOI:** 10.1016/j.mex.2021.101464

**Published:** 2021-07-21

**Authors:** Yichen Wu, Nan Zhang, Charles-François de Lannoy

**Affiliations:** Department of Chemical Engineering, McMaster University, Canada

**Keywords:** Boreal peats, Zinc chloride, Chemical activation, Pyrolysis, Isotherm, Kinetics

## Abstract

A fast, facile and one-pot chemical activation method was used to develop porous carbons with high surface area and excellent phenolic micropollutant adsorption performance from renewable precursors. This method was applied to three precursors: naturally abundant, but often underestimated wildfire-damaged boreal peats, corn starch, and cellulose. Porous carbon formation was accomplished through precursor impregnation with ZnCl_2_ powder and their simultaneous pyrolysis under inert N_2_ flow at 400 or 600 °C for 1 h. The maximum adsorption capacities of these bio-sorbents towards a model contaminant, *p*-nitrophenol, in simulated wastewater were equal to or superior than using a commercial activated carbon (CAC), Norit GSX (> 530 mg/g) over wide initial concentration ranges (20–2000 mg/L). *p*-nitrophenol adsorption best fitted Freundlich and Redlich-Peterson isotherms, suggesting multilayer chemisorption. Low concentration *p*-nitrophenol (20 mg/L) adsorption into the bio-sorbents was rapid in the first 4 h, and could reach high removals (> 98%). The method presented here yielded bio-sorbents with similarly high adsorption performance regardless of the precursor type, while avoiding energy-intensive processing steps during sorbent production. This study gives a useful alternative for manufacturing new sorbents from other upcycled carbonaceous and/or bio-based materials to remove micropollutants and heavy metals.

• Fast, single-step chemical activation for manufacturing bio-based porous carbons.
• Efficient adsorption towards aqueous phenolic micropollutant from batch studies.
• A competitive substitute of charcoal activated carbons for water purification.

## Graphical Abstract

**Figure:**
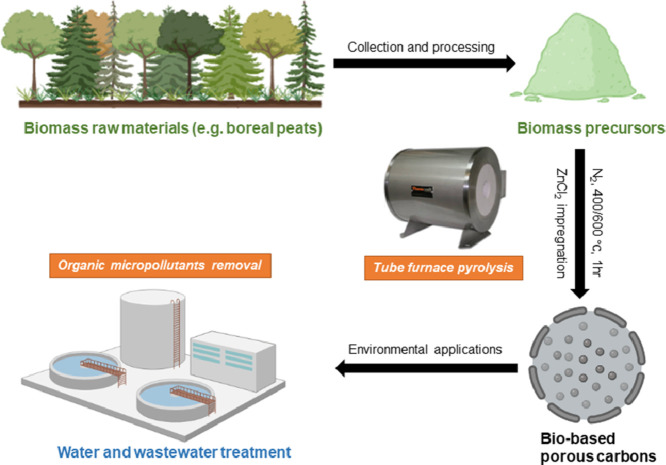

Specifications Table

**Table:** 

Subject Area:	Environmental Science
More specific subject area:	Bioresource technology, resource recovery, adsorption, wastewater treatment
Method name:	Zinc chloride activation for manufacturing biomass-based porous carbons
Name and reference of original method:	N/A
Resource availability:	N/A

## Background

Adsorption has been used for decades in water and wastewater treatment industries because of its simple design, ease of operation, and effectiveness, *i.e.,* the rapid removal of various inorganic and organic contaminants. Activated carbons (ACs), known to possess high surface areas, are maturely used in adsorption processing units. However, utilization of nonrenewable precursors, such as coals, along with traditional energy-consuming activation methods, cannot guarantee cheap manufacture in large quantities. Over the past several decades, production of bio-based sorbents from cheap, renewable agricultural and forestry wastes, and biowastes have been sufficiently investigated for the removal of varieties of aqueous contaminants, including pharmaceuticals [1,2], pesticides [3], phenols [4], dyes [5] and natural organic matter [6]. However, not all these precursors are suitable for industrial-scale production. For instance, fruit and vegetable residues usually require multistep processing [3,^4^,7], and face low output during treatment, storage and transportation. Agricultural byproducts can be obtained in larger quantities during crop harvesting and agro-industrial processing, but their supply is highly regional and seasonal. For instance, rice husk can be readily acquired by rice milling, but it is only available in the harvesting seasons in major rice-grown countries, such as China. Moreover, bio-based sorbents often neither achieve the high surface area nor the adsorptive performance of commercial CACs [8]. Furthermore, synthesis methods need to be improved. Various approaches have been developed to make bio-based sorbents, such as pyrolysis [2], carbonization and activation [1,4], and hydrothermal reaction [2,9]. These methods typically require relatively high temperature, long reaction time and multiple operating steps. Based on these limitations, the use of sustainably obtained biomaterials for conversion into efficient bio-based sorbents using rapid and facile synthesis methods will significantly benefit water treatment industries.

In the present study, we provide a feasible solution to address these challenges through utilizing the wildfire-damaged peats from the vast boreal regions of North America to produce porous carbons. Boreal peatland peats are organic soils, which serve important regional ecological functions, but have long been underestimated for environmental applications. Annual generation of surface peats could be considerable, as boreal peatlands cover 4,000,000 km^2^ of terrestrial area in North America and Eurasia. These peats are also a large reservoir for terrestrial carbon. Boreal peats suffer from frequent peat fires and droughts, which are becoming more frequent due to climate change [10]. Damaged surface peats contribute to water runoff, have been shown to impact the water quality of downstream communities, and can impede the regrowth of valuable boreal forests after forest fires. Damaged peats, however, represent an opportunity to harness the energy of forest fires for converting widely available organic material into precursors for bio-sorbents. We herein report the facile conversion of wildfire-impacted boreal peats, along with typical peat constituents (starch and cellulose), into highly porous bio-sorbents. This method involved *in-situ* ZnCl_2_ chemical activation, which only required moderately high temperature (400 or 600 °C) with a short treatment duration (1 h). Applications of the as-prepared porous carbons for environmental pollution control, *i.e.*, the adsorption of a notorious micropollutant, *p*-nitrophenol from simulated wastewater was also discussed to reveal both their mechanisms of adsorption and their great potential for water treatment.

## Method details

### Collection and treatment

**Fig. 1:**
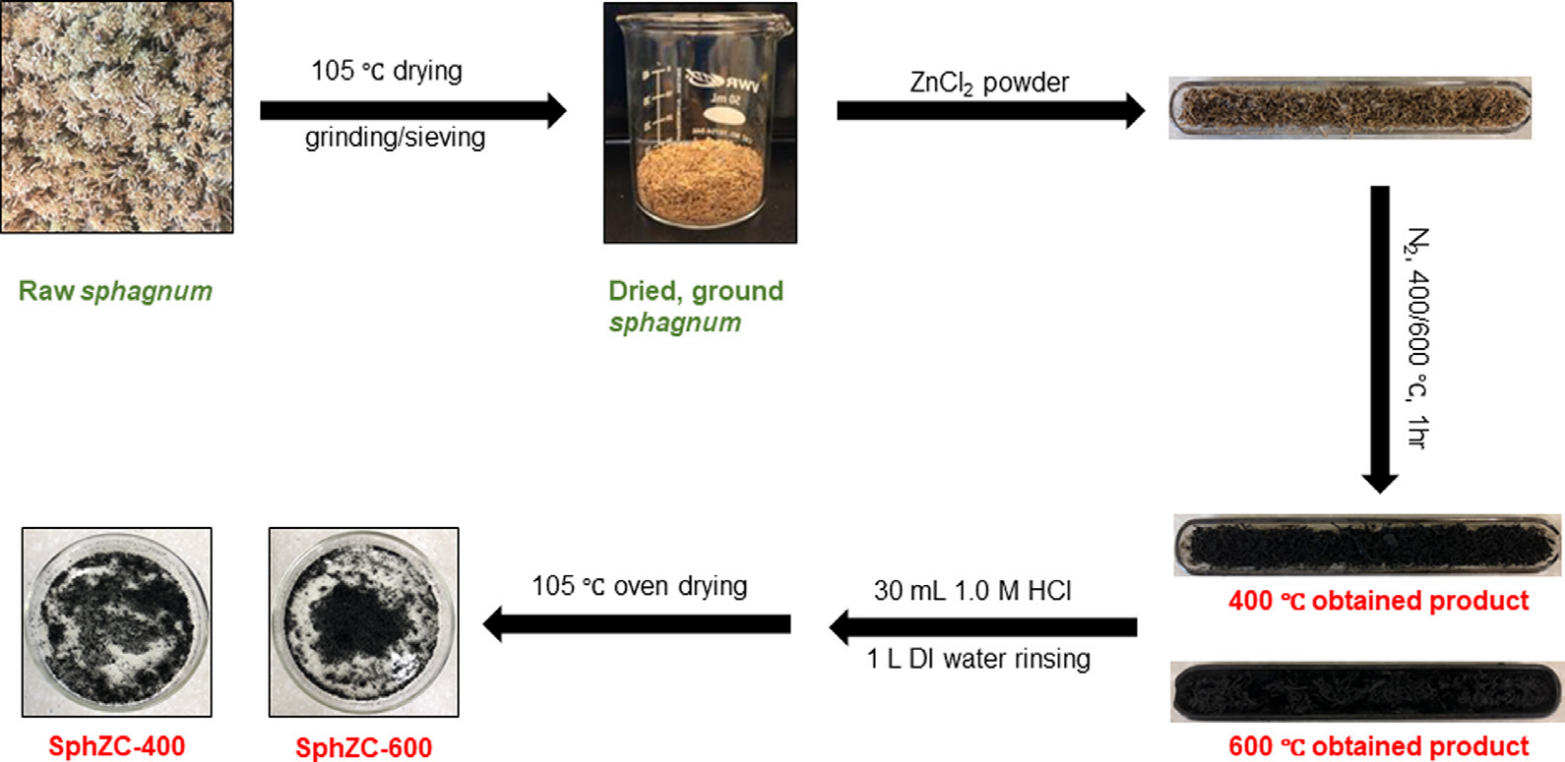
A schematic presentation of main processing steps for obtaining *sphagnum* derived porous carbons.

Unburned surface boreal peats, including *feather moss* and *sphagnum* peats, were cored in PVC columns (H = 15 cm) from the Pelican Mountain Research Site (55°36’ N, 113°35’ W), Alberta, Canada. These two species were chosen as precursors because they are the most dominant moss species in the boreal spruce peatlands. Large twigs, plant roots and wood debris were manually removed. *Sphagnum* peat sampling and processing steps are shown in Fig. 1 as an example. Live *feather moss* was easily separated from underlying decomposed peats. *Sphagnum*, however, had no clear boundary with the underlying decomposed peat. Hence, the entire 15 cm *sphagnum* peat soils were picked as the feedstock. *Feather moss* and *sphagnum* samples were dried at 105 °C overnight in an oven to constant weight, ground and sieved (Fisher, dia. 8 inch) to less than 2 mm size. Post-burn peats were simulated through heating *feather moss* and *sphagnum* samples in a muffle furnace (Fisher Scientific, USA) at 300 °C for 30 min. 300°C was selected because this was within reasonable range of temperatures that peat fires occur (*i.e.*, 250–300 °C) [10]. Moreover, we picked corn starch (Sigma-Aldrich) and cellulose (Sigma-Aldrich) as precursors, due to their low cost and abundance in North America, as well as their potential to partly mimic peat constituents, *i.e.*, polysaccharidic and lignocellulosic substances. Corn starch and cellulose were also oven-dried at 105°C overnight to constant weight. Preliminary experiments indicated that raw starch swelled significantly upon heat treatment and formed tar products with very low surface area after chemical activation. However, a previous study obtained high surface area spherical carbons through KOH activation from heat-stabilized cassava starch [11]. Here a similar approach was taken, where corn starch was first stabilized in a muffle furnace at 220 °C for 16 h to eliminate starch chain hydroxyl groups and melt the crystallites. The stabilized starch was further pulverized by a coffee grinder, then passed through a 2 mm sieve. Cellulose was not further treated prior to the activation. The biomass feedstocks, including unburned *feather moss*, unburned *sphagnum*, 300 °C lab-burned *feather moss*, 300 °C lab-burned *sphagnum*, 220 °C heat-stabilized corn starch and cellulose, were respectively labelled as Fm, Sph, BFm-300, BSh-300, BCS-220 and CL.

### Chemical activation

Fm, Sph, BFm-300, BSph-300, BCS-220 and CL were respectively impregnated with ZnCl_2_ (anhydrous, ≥97%, Sigma-Aldrich) at a mass ratio of 1:3. All impregnated samples were well-stirred by a glass rod before chemical activation. Certain masses of these mixtures were loaded onto a quartz boat (100 × 15 × 75 mm), which was then transferred into a quartz tube. Before the pyrolysis, the tube furnace (Thermocraft Inc., USA) was preset to 200 °C. Samples were heated up to 400 °C at a rate of 7 °C/min and maintained for 1 h, or 600 °C at a rate of 13 °C/min and maintained for 1 h, under constant nitrogen flow (100 mL/min). After the reaction, the 400 °C treated samples were directly removed from the chamber, whereas the 600 °C treated samples were allowed cooling in the chamber for 20 min under N_2_ flow before removing. The products were washed with 30 mL 1.0 M HCl (Sigma-Aldrich) followed by 1 L DI water until constant effluent pH, followed by drying overnight at 105 °C in an oven. The obtained porous carbons were respectively labelled as FmZC-T, SphZC-T, BFmZC-T, BSphZC-T, BCSZC-T and CLZC-T, where T (°C) refers to the activation temperature. The detailed physicochemical characterizations of the as-prepared biomass porous carbons were described in our previous publication [8].

### *p*-nitrophenol adsorption

#### Batch adsorption

Batch adsorption experiments were performed on a multiposition stir plate (BT lab systems, USA) by continuous stirring of synthetic phenolic wastewater at 20 °C with addition of sorbents. To keep the pH constant for whole adsorption periods, the synthetic wastewater was prepared at phosphate buffer solutions. Briefly, every 1.165 g NaH_2_PO_4_·H_2_O and 3.097 g Na_2_HPO_4_·7H_2_O were dissolved in 1 L DI water to afford the phosphate buffer solution (pH = 7, Molarity=20 mM). Preliminary tests indicated that this buffer condition was promising, as a pH deviation of only less than 0.3 was found for the tested *p*-nitrophenol concentration ranges (20–2000 mg/L) before or after adsorption. The sorbed *p*-nitrophenol on various biomass porous carbons was calculated by the following equation.

(1) $$q_{t}=\frac{V(C_{0}-C_{t})}{m}$$

where $q_{t}$ is the adsorption capacity at time t (mg/g); $C_{0}$ and $C_{t}$ are the *p*-nitrophenol concentration at initial and a certain time t, respectively; V is the solution volume (L); m is the sorbent weight (g). When the adsorption reaches equilibrium, the equilibrium concentration of *p*-nitrophenol can be referred to $C_{e}$ (mg/L), such that the equilibrium adsorption capacity $q_{e}$ (mg/g) can be expressed as follows.

(2) $$q_{e}=\frac{V(C_{0}-C_{e})}{m}$$

The concentration of *p*-nitrophenol was determined by UV–vis spectrometry performed on a DU800 UV–vis spectrophotometer (Beckman Coulter, USA). Before adsorption experiments, calibration curve for *p*-nitrophenol was established in the buffered solution, and a maximum absorbance wavelength at 400 nm was applied for the measurements.

#### Adsorption isotherm

25 mL buffered *p*-nitrophenol simulated wastewaters at various concentrations (20–2000 mg/L) were transferred into 50 mL conical flasks, followed by addition of 10 mg sorbents and stirring at 200 rpm at 20 °C for 24 h to allow adsorption equilibrium. Suspensions were afterwards filtered by 25 μm PVDF membranes, and diluted at proper magnifications (*e.g.*, 4, 20, 50, 100 times) if necessary, for UV–vis spectrophotometer measurements. Freundlich, Langmuir and Redlich-Peterson isotherms were used to model *p*-nitrophenol adsorption behaviors. These isotherm types are expressed as follows.

(1) Freundlich isotherm

(3) $$q_{e}=k_{F}C_{e}^{1/n}$$

(2) Langmuir isotherm

(4) $$q_{e}=\frac{q_{m}k_{L}C_{e}}{1+k_{L}C_{e}}$$

(3) Redlich-Peterson isotherm

(5) $$q_{e}=\frac{k_{RP}C_{e}}{1+\alpha_{R}C_{e}^{\beta }}$$

where $k_{L}$ (L/mg) is the Langmuir constant; $k_{F}$ ($\left(mg/g\right)\cdot {\left(L/mg\right)}^{1/n}$) is the adsorption capacity; $k_{RP}$ (L/g) is the Redlich-Peterson constant; 1/n is an empirical constant; $\alpha_{R}$ and β are constants; $C_{e}$ (mg/L) is the equilibrium *p*-nitrophenol concentration; $q_{e}$ (mg/g) and $q_{m}$ (mg/g) are the equilibrium adsorption capacity and maximum adsorption capacity, respectively.

**Fig. 2:**
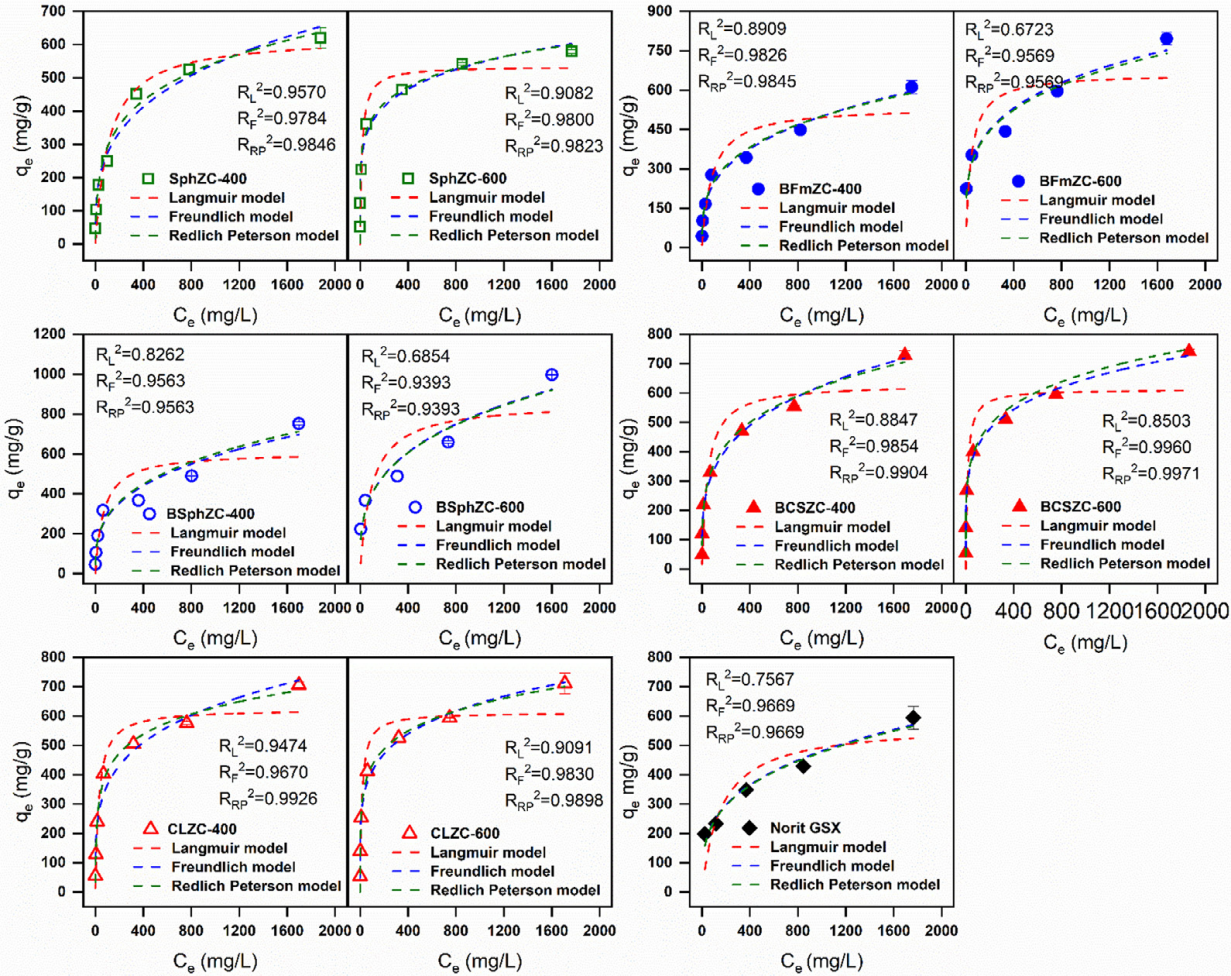
Adsorption isotherms of *p*-nitrophenol onto selected bio-based porous carbons and Norit GSX at 20 °C. $R_{L}^{2}$, $R_{F}^{2}$ and $R_{RP}^{2}$ represent the fitted determination coefficient of Langmuir, Freundlich and Redlich Peterson model, respectively.

Representative modeled adsorption isotherms were plotted in Fig. 2 as well as described in our previous publication [8]. The Langmuir model suggests monolayer adsorption, assuming identical adsorptive sites with unform energy level [12]. The Freundlich model by comparison is based on multi-layer adsorption involving chemisorption mechanism onto heterogeneous surfaces [12]. The Redlich-Peterson model combines both the Langmuir and Freundlich models which can be applied in homogeneous or heterogeneous systems [13]. This hybrid model approaches ideal monolayer adsorption at low sorbate concentrations, but approximates multilayer adsorption at high sorbate concentrations [13]. By fitting data into these models, a mechanistic understanding of sorbent-sorbate interaction can be obtained. Freundlich model and Redlich-Peterson model fittings have the highest *R*^2^ values, suggesting that *p*-nitrophenol adsorption involved multilayer chemisorption.

#### Kinetics

25 mL 20 mg/L buffered *p*-nitrophenol simulated wastewaters were dosed with 10 mg sorbents and stirred on a multiposition stir plate at 200 rpm at 20 °C for different time durations (0–4 h). Suspensions were then taken and filtered by 0.2 μm PVDF membranes, for UV–vis spectrophotometer measurements. Preliminary studies indicated that adsorption of 20 mg/L p-nitrophenol for 4 h on each sorbent can yield a removal efficiency of greater than 98%. After 4 h, the adsorption continued to occur but at an extremely slow rate. Therefore, the adsorption capacity at 24 h was considered as the equilibrium adsorption capacity. Pseudo-first-order model, pseudo-second-order model [14] and intra-particle diffusion model [15] were used to evaluate the adsorption kinetic behavior. The equations can be expressed as follows.

(1) Pseudo-first-order model

(6) $$\ln\left(q_{e}-q_{t}\right)=\ln q_{e}-k_{1}t$$

(2) Pseudo-second-order model

(7) $$\frac{t}{q_{t}}=\frac{1}{k_{2}q_{e}^{2}}+\frac{t}{q_{e}}$$

(3) Intra-particle diffusion model

(8) $$q_{t}=k_{d}t^{1/2}+C$$

where $k_{1}$ ($\min^{-1}$) is the pseudo-first-order rate constant; $k_{2}$ ($g\cdot mg^{-1}\cdot \min^{-1}$) is the pseudo-second-order rate constant; $k_{d}$ ($mg/g\cdot \min^{0.5}$) is the intra-particle diffusion rate constant; $q_{e}$ (mg/g) and $q_{t}$ (mg/g) are the adsorption capacity at equilibrium and at time t (min); C is a constant.

**Fig. 3:**
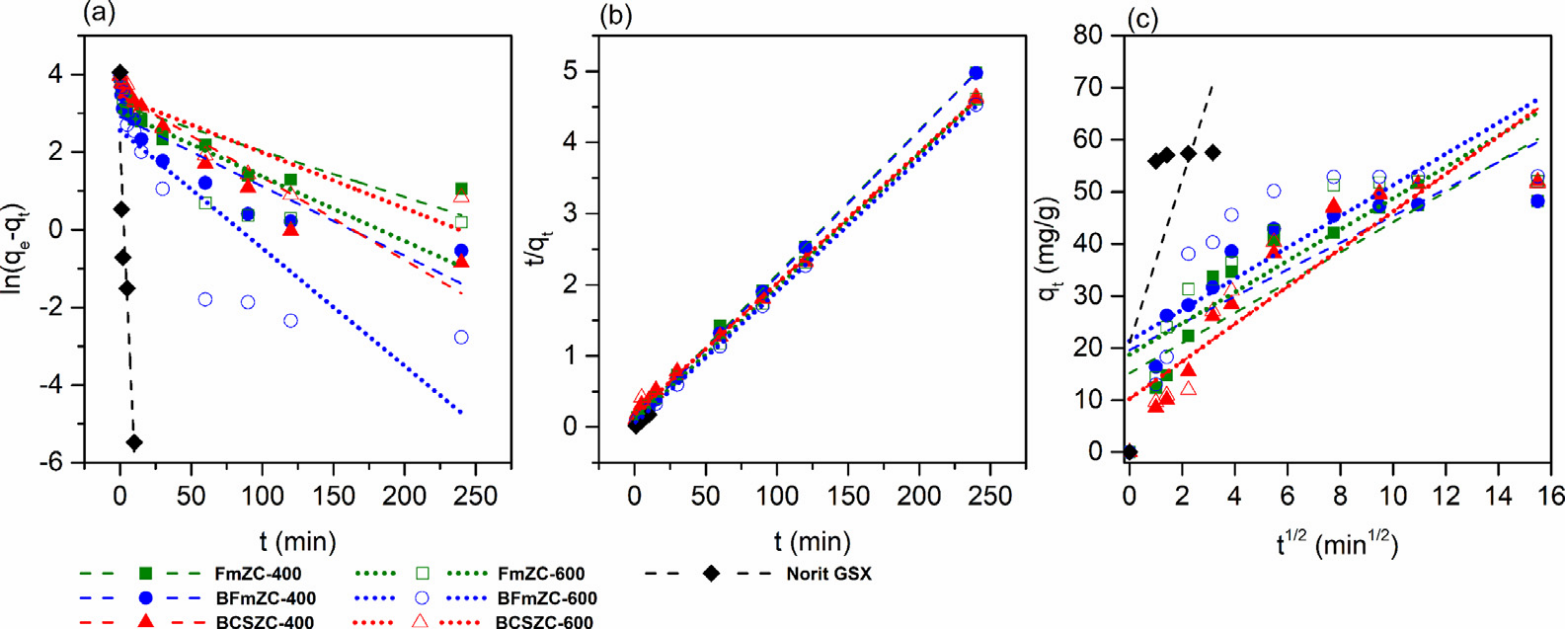
Adsorption kinetics modeling of *p*-nitrophenol onto selected bio-based porous carbons and Norit GSX at 20 °C in linearized forms. (a) Pseudo-first-order model; (b) Pseudo-second-order model; (c) Intra-particle diffusion model.

Linear fitting of kinetic models for selected sorbents were plotted in Fig. 3. It is clear that a pseudo-second-order model was the optimal fit for the kinetic adsorption data, as indicated by the high *R*^2^ values (> 0.99), which were much higher than that of the pseudo-first-order model (*R*^2^ < 0.91) and the intra-particle diffusion model (*R*^2^ < 0.84). This indicated that *p*-nitrophenol adsorption onto the activated carbons was mainly controlled by available adsorptive sites and adsorbate concentration in the system [16]. Worth noting, the scatter points of the intra-particle diffusion model (Fig. 3c) for the selected bio-based porous carbons had gradually decreased slopes, suggesting the adsorption can be described by multiple adsorption rates, which accorded with the decreased adsorption rate after 4 h in the kinetic test.

## Conclusion

This research demonstrates a simple, one-step chemical activation method using ZnCl_2_ as an activation agent to obtain high surface area carbon-based sorbents from wildfire-impacted boreal peats, corn starch and cellulose precursors. The feedstocks can be easily-accessed in large quantities in North America, and their pretreatments prior to pyrolysis are simple and accessible to nearly all labs and most industrial producers. We also investigated the adsorption of *p*-nitrophenol onto the sorbents and fitted both Freundlich and Redlich-Peterson isotherms to these adsorption curves. The details of these fits and the equations used for these fits are presented herein. We suggest that fitting multiple isotherms is important for probing the adsorptive mechanism. Furthermore, analyzing both steady-state adsorption and kinetic adsorption is important for understanding the efficacy of these sorbents in comparison to commercial ACs. We suggest that both steady-state and kinetic studies should be conducted, along with proper model fitting, for all bio-based sorbents being developed in the field so as to establish proper comparisons across this wide range of materials. In this way advantages and disadvantages of new sorbent materials can be sufficiently evaluated. These peat-based sorbents had excellent adsorption performance towards a typical phenolic model contaminant, *i.e., p*-nitrophenol, in simulated wastewater, comparable or higher than that obtained from a common market-sale CAC, Norit GSX. Because the manufacturing route for the biomass sorbents was easy and efficient, we encourage that other carbonaceous materials, such as agricultural wastes, be collected and treated for producing new bio-based sorbents following a similar approach. In addition, attempts to treat other organic molecules (*e.g.*, azo dyes and pharmaceuticals), and heavy metals can be made on the basis of these biomass sorbents in future studies.

## Declaration of Competing Interest

The authors declare that they have no known competing financial interests or personal relationships that could have appeared to influence the work reported in this paper.
